# The Dat Project, an open and decentralized research data tool

**DOI:** 10.1038/sdata.2018.221

**Published:** 2018-10-23

**Authors:** Danielle C. Robinson, Joe A. Hand, Mathias Buus Madsen, Karissa R. McKelvey

**Affiliations:** 1 Code for Science & Society, Portland, OR 97214, USA; 2 The Dat Project, Portland, OR 97214, USA; 3 Beaker Browser, Portland, OR 97214, USA; 4 Digital Democracy, Portland, OR 97214, USA

**Keywords:** Software, Research data, Data publication and archiving

## Abstract

Today’s scientific data are primarily stored and accessed via centralized Web-based infrastructure. Centralization has advantages but also carries risks such as link rot and content drift, which can hinder scientific progress. It is time to ask whether traditional, centralized Web architecture aligns with scholarly priorities and values, and to collaboratively move towards new approaches that do.

## Comment

Information is increasingly shared, stored, and accessed in exclusively digital forms. Ensuring stable, free access to digital information is therefore one of the most important challenges of our time. Access, preservation, and reusability of scientific data are influenced by factors from data collection through publication. As scholars and the larger system of scholarly record begin to recognize data as a citable research object, it becomes critical to manage and archive data-driven research outputs with long term access and reuse in mind^1,2^. The research community needs tools that fit into researcher workflows and prioritize data permanence, while supporting flexible workflows in complex computing environments. We propose leveraging decentralized computing technology that prioritizes data authenticity, discoverability, access, and permanence with the Dat Protocol.

Our team has been developing Dat, a decentralized data sharing technology, with a focus on scientific use cases. Over the past year, we have been applying this software to real-world research problems. The Dat in the Lab project (https://blog.datproject.org/tag/science/) has shown the critical need for research tooling built around Dat’s core features: decentralized file sharing, automatic file versioning, and secure data backup. Some of these ideas exist in other data management platforms. Dat uniquely combines these necessary features in tools that work with existing researcher workflows. With support from the Gordon and Betty Moore Foundation (https://www.moore.org), and in partnership with UC Curation Center (UC3, https://www.cdlib.org/uc3/), a program of California Digital Library (https://www.cdlib.org/), we have worked with researchers to identify places where decentralized tooling can support research workflows. Dat solves basic research data management problems, by automating data synchronization and versioning; research labs can use these tools for sharing large datasets across institutional boundaries. Additionally, we’ve worked with labs to develop new use cases, such as coordinating and syncing complex compute environments across different high performance computing clusters. Through this work with researchers, we have shown that decentralized computing tools can improve scientific data management. As the next step in this work, we are piloting a cooperative decentralized data preservation network with the Internet Archive (https://archive.org/), San Diego Supercomputer Center (http://www.sdsc.edu/), and California Digital Library.

## A brief history of the benefits and costs of data centralization

All technology is imbued with the values and biases of its creators^3^. The data storage and preservation systems available to researchers today are no exception. Most of today’s scientific artifacts are digital objects preserved and accessed via the Web. The architecture and incentive structures of the Web impact how research data are preserved and distributed (just as incentives for scientists impact data publication)^4^. The Web has made information and scholarship easier to access today than at any other time in human history.

While the Web has the potential to enable full open access to knowledge, the code that powers the Web is not built for that. Instead, the Web uses a centralized data model optimized for use by commercial organizations^5^. In other words, today’s Web values the access and voices of people who are valuable to corporate interests. Scientific publications and other research outputs are preserved as digital objects, including software dependencies, compute environments, and data. Many institutions now offer stewardship of scientific outputs, taking custody of data for long term storage and preservation. This removes the burden from researchers to host data but places the burden of storage on the institution or a third party preservation entity. Data tend to be stored as static objects, and are ideally FAIR^6,7^. However, link rot (when data no longer exist at a link) and content drift (when the data are changed) — collectively called reference rot — are still issues for scholars in data intensive fields^8,9^.

Most data services available to researchers today are centralized. At a centralized data repository or other online data service, data are stored at a single physical location, such as a data center. Access to those data are controlled by the owner of that location. Data sharing, transfer, and collaboration must take place through that location. Although the term “cloud” implies lack of centralization, access to data centers are massively centralized. By creating a single access point, centralized services improve discoverability and access. Considered in isolation, a well-managed information silo is a stable place for data storage. Pragmatically, centralized services have also been beneficial to scholars and institutions because they are cheaper and easier to manage than in-house data storage services.

The proliferation, however, of centralized services has created a landscape of information silos with limited interoperability, reducing the reach and impact of data. Access to data is controlled by the service that holds the data, and may be granted freely or restricted based on institutional affiliation. While there are more options than ever before for scholars who want to share, preserve, or access data — discovery and access across silos is limited. As the global volume of data increases, will this model scale? A siloed, centralized data preservation model benefits entities that manage, monetize, and gate-keep access to information. This model disincentivizes cooperative infrastructure for sharing information and does not prioritize data access or preservation.

It is easy to use the Web for data discovery, access, and preservation without pausing to consider how the architecture of the Web influences that process. The bias’ and priorities’ of the Web’s architects have created the Web we interact with today. As an example, early online business models lead to today’s commodification of content and attention. Today, we are accustomed to online infrastructure that requires a form of payment (money, attention, data) for access to information^10^. For knowledge to be disseminated globally, the Web must be reimagined as a space that prioritizes access over profit. Considering the limitations of the Web is the next step towards greater access to scholarly work and knowledge for all. We encourage institutions, libraries, and scholars to consider whether the values of the existing system are in line with their institutional (and personal) mission and goals.

## Properties of the Dat Project that aid knowledge dissemination

Decentralized models rethink how data are owned, preserved, and accessed. Decentralized models include peer-to-peer (P2P) and other distributed systems^11^. We will focus on Dat, the P2P file sharing protocol, and discuss its potential to impact how scientific data are managed and shared. We believe that introducing decentralization at an infrastructural level will allow existing silos (institutional data repositories, third party data preservation platforms) to share information, making data easier to access, improving redundancy, and forming the basis of a cooperatively run data preservation network.

While alternatives to centralized models, like P2P and other decentralized technologies, are a part of the Web’s history and have been used to circumvent centrally controlled systems^12^, the majority of the Web remains centralized. Although academic outputs like data and publications are not intended to be commercial, their dissemination online is shaped by the Web’s structure and business models. New decentralized approaches are impacting how the Web is built by provide alternative models for knowledge dissemination. While many decentralized systems allow for easier sharing of data, we will focus on the specific properties of Dat moving forward.

Dat is a new P2P hypermedia protocol, built on existing internet technologies, to allow people to share information in a decentralized network. In other words, it allows users to handle publishing, dissemination, and backup of information across a network of computers, rather than from a central server. Dat began as a grant-funded open source project to improve the accessibility of data in science. Dat continues to be a mission-driven project, with contributors working in research, new media, government, and journalism.

Dat was developed to distribute and archive datasets of any size. When a folder is tracked with Dat, it creates a unique persistent identifier for that package of data (whatever is in the folder). This unique identifier allows the folder to contain dynamic content while keeping the same identifier. Additionally, Dat tracks changes to the contents of the folder (i.e. version control) with a transparent change-log. Any reader can view the change-log, see early versions of the dataset, or sync the folder to always have the latest version. For more on how Dat works, see the whitepaper^13^ or visit https://docs.datproject.org.

At their fundamental level, decentralized systems distribute data across a network of linked participants^14^. In Dat, objects stored in the network are authenticated by their creator and include a transparent log of the object’s history. Objects can be downloaded, their integrity verified, and stored locally for offline use. Together, these principles improve the availability of objects by allowing verified copies to be stored in many locations. If the original author cannot maintain their copy, another entity can collect a verified copy and keep it accessible. This gives researchers and institutions the freedom to copy and archive datasets that are valuable to them, reducing link rot^14^. Dat networks are also useful in low-connectivity contexts, which are critical to any attempt at improving global dissemination of and access to knowledge.

Today, Dat is used by people across domains as building block for rethinking the way data are owned, shared, and preserved online. Examples include: Mapeo, an offline-friendly mapping software to support indigenous land rights (Digital Democracy, https://www.digital-democracy.org/mapeo/); Peer-to, an online art exhibition only available on the p2p web (https://peer-to.peer-to-peer-web.com/); a peer-to-peer browser, Beaker Browser (https://beakerbrowser.com/), and its community of affiliated projects and creative tools; and, a desktop scientific publication library, ScienceFair (http://sciencefair-app.com/). Each of these applications solves problems of information access and integrity with the Dat protocol. By collaborating with users across domains, the Dat team is focusing on solving real-world problems with P2P approaches. The global community of people working with Dat are creating new models for sharing of data and forging new paths for distributed information access.

## Reimagining data preservation at libraries with decentralized models

Librarians, technologists, and scholars are developing and managing systems to preserve humanity’s growing knowledge base. The DataRescue initiative highlighted the instability of Web-based storage of research data^15,16^. As Laurie Allen said, “the internet is a terribly unstable way to keep information available”^15^. Decentralized technologies like Dat can change the web by ‘locking it open’ as a library of human knowledge truly accessible to all^17^. The Web is not designed for the scale of long term preservation of digital information that humankind is now experiencing. The Dat Project is aiming to solve this problem by using P2P technology to democratize access to data.

Centralized data storage systems can only preserve what they hold in their servers. This model requires custody to provide access. Data custody becomes increasingly expensive and difficult to manage as data volumes increase; it also places more burden on website maintainers to keep links and locations updated. Stephen Abrams asks the question, “can we replace custody with easy access?”^18^ The idea of “preservation in place” where libraries bring “preservation services to the content” transforms the requirements for data preservation^19^. In other words, is knowing where data are, and trusting the preservation standards of that location, equivalent to (or better than) custody? Can we reduce the burden on institutions to own everything with a mandate to know where data are and how many verified copies exist?

In a decentralized model, custody is not required for access. In a Dat network, custody is replaced by access to a verified copy. Data then live in a network of linked institutions. Decentralized models make preservation in place technically feasible and interoperable with existing data preservation silos. Information on data collections can be shared between entities today. A decentralized network takes this a step further by automating the sharing of information on collections, allowing access to other entities’ digital objects, and encouraging the creation of verified copies (note that a common metadata standard is critical). In this way, data can be redundantly stored in multiple locations, reducing the cost burden on each entity. When access is needed, data are downloaded from the most convenient copy.

With our partners, Internet Archive, San Diego Supercomputer Center (http://www.sdsc.edu/), and California Digital Library, we are building a pilot of a decentralized storage network on top of their existing data preservation systems^20^. Our collaboration will give us the opportunity to work directly with stakeholders to develop decentralized information sharing tools that are interoperable with existing systems and easy for librarians and researchers to use.

Our goal is to spread verified copies of data across many institutions, ensuring open access and reducing long-term costs for libraries. Librarians will see which institutions are currently storing data, as well as metrics on data usage. By having access to the “health” of content, libraries can make informed decisions for research data management and institutional curation. Because Dat’s networking and information sharing occur at an infrastructure level, Dat can prioritize interoperability and compatibility with existing systems (eg: DASH), and support proliferation of common metadata standards (eg: DataCite), to support cost savings for libraries.

The Dat Protocol creates opportunities for researchers, librarians, technologists and other stakeholders to rethink data management at all levels, from the individual researcher to institutions^21^. Rethinking the way data are stored and accessed online with decentralized data models will improve research reproducibility and benefit the global scientific enterprise. Ultimately, our goal is to make it easy for researchers to share and consume data, and for society at large, including scientific institutions, to collect and preserve it. Join us online at https://datproject.org to learn more and follow our work.

## Additional information

**How to cite this article:** Robinson, D. C. *et al*. The Dat Project, a new approach to support data preservation through decentralization. *Sci. Data*. 5:180221 doi: 10.1038/sdata.2018.221 (2018).

**Publisher’s note:** Springer Nature remains neutral with regard to jurisdictional claims in published maps and institutional affiliations.
